# Electron beam irradiated silver nanowires for a highly transparent heater

**DOI:** 10.1038/srep17716

**Published:** 2015-12-07

**Authors:** Chan-Hwa Hong, Seung Kyu Oh, Tae Kyoung Kim, Yu-Jung Cha, Joon Seop Kwak, Jae-Heon Shin, Byeong-Kwon Ju, Woo-Seok Cheong

**Affiliations:** 1 Electronics and Telecommunications Research Institute, Yuseong-gu, Daejeon 305-700, South Korea; 2 Display and Nanosystem Laboratory, College of Engineering, Korea University, South Korea; 3 Department of Printed Electronics Engineering, Sunchon National University, Jeonnam 540-742, South Korea

## Abstract

Transparent heaters have attracted increasing attention for their usefulness in vehicle windows, outdoor displays, and periscopes. We present high performance transparent heaters based on Ag nanowires with electron beam irradiation. We obtained an Ag-nanowire thin film with 48 ohm/sq of sheet resistance and 88.8% (substrate included) transmittance at 550 nm after electron beam irradiation for 120 sec. We demonstrate that the electron beam creates nano-soldering at the junctions of the Ag nanowires, which produces lower sheet resistance and improved adhesion of the Ag nanowires. We fabricated a transparent heater with Ag nanowires after electron beam irradiation, and obtained a temperature of 51 °C within 1 min at an applied voltage of 7 V. The presented technique will be useful in a wide range of applications for transparent heaters.

Transparent heaters are commonly used in such applications as vehicle windows, military ground based vehicles, periscopes, and public information displays. Especially, the future direction of transparent heaters focuses anti-fogging windshields, mirrors, and displays that ensure the fast response of electronic devices under various environmental conditions. Indium tin oxide (ITO) film has been widely used as a transparent film heater in the industry^1^. However, ITO exhibits a slow thermal response and requires complicated fabrication processes. Moreover, its brittle ceramic properties and expensive vacuum deposition process are limitations to its further progress^2^. Thus, various indium-free transparent conductive materials have been extensively investigated as alternatives to ITO: conducting polymers^3^,^4^ graphene^5^,^6^, carbon nanotubesv^7^,^8^, and several conducting oxides, such as Al:ZnO^9^, Ga:ZnO^10^, and F:SnO^11^. Among these, silver nanowires (AgNWs) have recently attracted substantial interest as a transparent conducting material due to their transparency, low resistivity, and non-vacuum processes^12^,^13^,^14^,^15^,^16^. However, some fundamental issues must be resolved, including surface roughness, week mechanical adhesion force, and highly haze properties^17^. In particular, researchers found that it was necessary to achieve a higher conductivity in AgNWs to create the highly generated transparent heater. Many studies have reported on AgNW electrodes achieving high levels of performance for the devices^18^,^19^. Recently, E. C. Garnett *et al.* reported a novel method for high quality AgNWs. They suggested that a plasmonic welding technique provide extreme heating at the junctions of AgNWs by illumination from a tungsten-halogen lamp, the resulting welded AgNWs had higher conductivity without broken wires^20^. However, that paper indicated that the illumination must exceed a temperature of 150 °C, and that temperature is not acceptable for flexible substrates.

To overcome these problems, we investigated the electrical, optical and structural properties of AgNWs by electron beam irradiation with low temperature. The results demonstrated that electron beam irradiation significantly reduces the sheet resistance of AgNWs, which in turn enhances the heat generation behavior for transparent heater applications.

## Experiments

**Figure 1:**
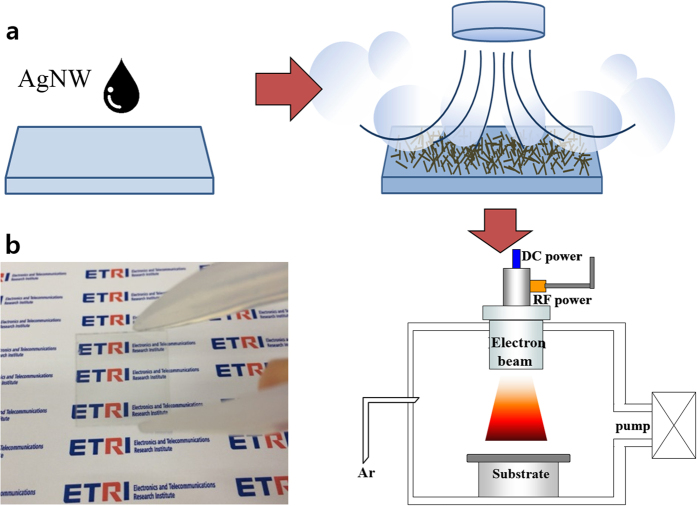
Schematic fabrication process of AgNW electrodes (**a**), and a fabricated AgNW thin film on glass with high transparency (**b**).

As shown in Fig. 1(a), AgNWs were coated on corning glass by a drop casting method. Then, the coated substrate was dried for 30 sec in wind at a temperature of 60–70 °C with velocity of 17 m/s. The typical diameter and length of the AgNWs in the dispersion were approximately 27–30 nm and 5–10 μm, respectively. The AgNWs were dispersed in isopropanol (IPA) with concentration of 0.15 wt%. After we made the AgNWs on glass, an electron beam (Infovion inc.) irradiated them for 0–180 sec with a RF power of 150 W and a DC power of 1.5 kV in a vacuum chamber. The electron beam source consisted of an inner RF (13.56 MHz) coil antenna and two grid electrodes for discharging Ar plasma and for collimating/accelerating the electron beam towards the substrate, respectively^22^. The sheet resistance of the AgNWs was measured using a four-point probe. The optical transmittance was measured in a wavelength range of 350–800 nm by UV-spectrophotometer. Surface morphology images of the samples were obtained using a field-emission scanning electron microscopy (FE-SEM). To explain the structural properties, the AgNW thin films were analyzed by high resolution transmission electron microscopy (TEM) and selected area electron diffraction (SAED) of the TEM image. Mechanical adhesive force was tested using 3M Scotch tape. The voltage source was directly connected to both ends of the Al foil electrode. The electrical current through the AgNW thin films was measured using a digital multimeter (Agilent, B1500a). The surface temperature and the thermal image were obtained by a thermal imaging camera (Flir, i3).

## Results and Discussion

**Figure 2:**
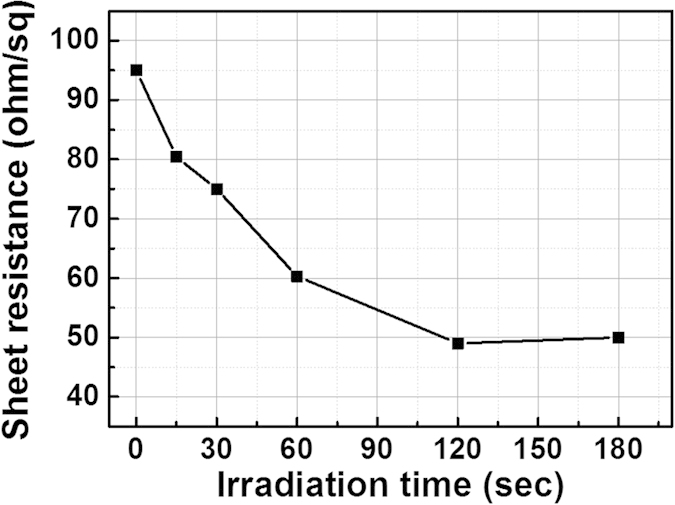
The sheet resistance of AgNW thin film as a function of electron beam irradiation time.

Figure 2 shows the variation of the sheet resistance of the AgNWs as a function of electron beam irradiation time. As the figure shows, with an increase in the electron beam irradiation time, the sheet resistance of the AgNWs decreased from 95 ohm/sq to 48 ohm/sq after electron beam irradiation for 120 sec.

**Figure 3:**
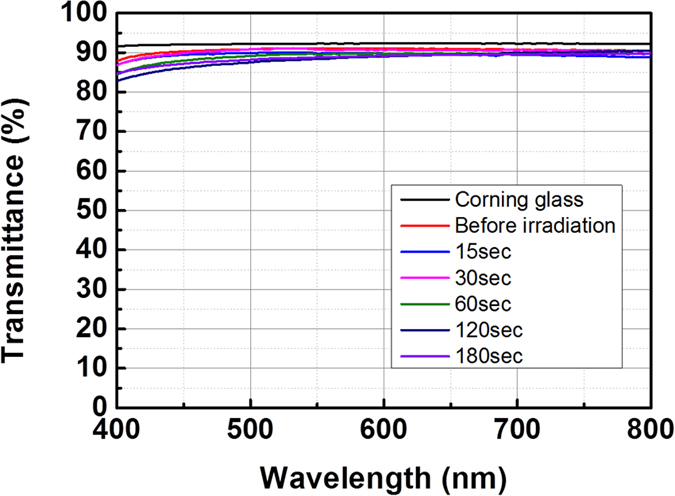
The transmittance of AgNWs with different electron beam irradiation times.

**Figure 4:**
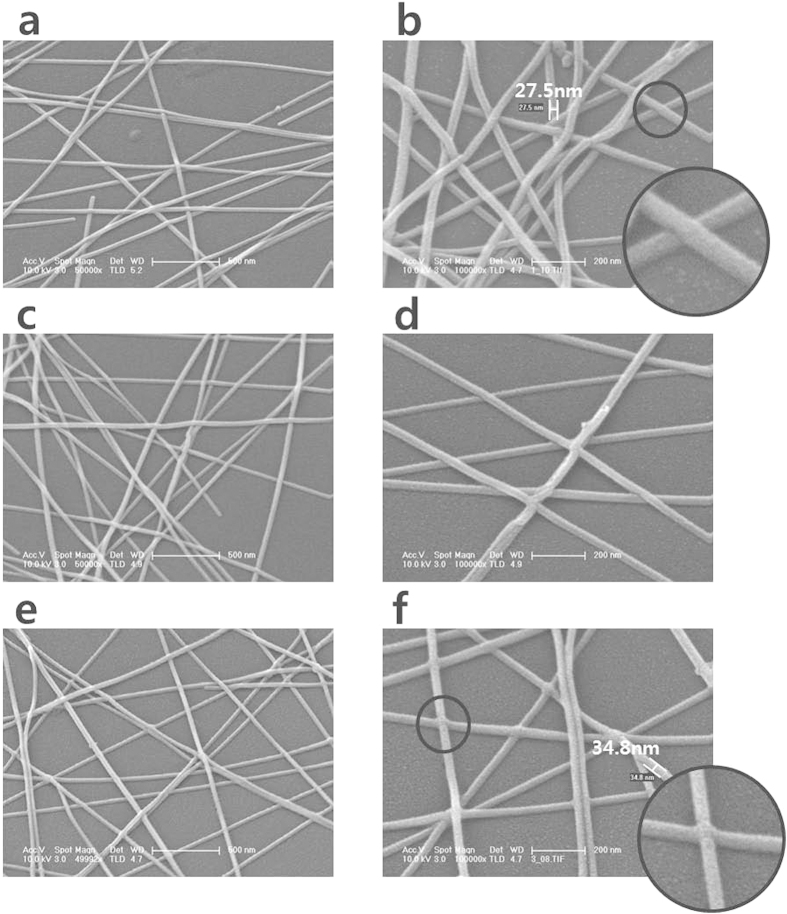
The SEM images of AgNW thin film before electron beam (**a,b**), after 30 sec electron beam irradiation (**c,d**), and after 120 sec electron beam irradiation (**e,f**).

Figure 3 shows the change of transmittance of the AgNW/Corning glass before and after the electron beam irradiation. As the figure shows, the transmittance (at 550 nm) of the AgNW/Corning glass system generally decreased from 91.1% to 88.8% after electron beam irradiation for 180 sec. Although the sheet resistance of the AgNWs dramatically decreased after electron beam irradiation, transmittances did not decrease much after electron beam irradiation. Moreover, the AgNWs maintained a high degree of transparency in the whole visible range, as Fig. 3 confirms. A picture of the AgNW/glass with a high degree of transparency after electron beam irradiation (120 sec) is given in Fig. 1(b). Figure 4 exhibits a scanning electron microscopy (SEM) image of AgNWs before electron beam irradiation (Fig. 4(a,b)), after electron beam irradiation for 30 sec (Fig. 4(c,d)), and after electron beam for 120 sec (Fig. 4(e,f)). The AgNWs appear stacked in all directions without soldering or sintering. The nano-soldering after electron beam irradiation at the nanowire junctions is clearly shown in Fig. 4(f). The dimensions of the AgNWs before electron beam irradiation, after electron beam irradiation for 30 sec, and after electron beam irradiation for 120 sec were 27.5 nm, 32.7 nm, and 34.8 nm, respectively. Thus, we can expect that although the increased dimensions of the AgNWs and junctions leds to slight decrease in the transmittance, the nano-soldering reduced the contact resistance of the AgNW networks dramatically at the nanowire junctions. These results enhanced the electrical properties of the AgNWs with a high degree of transparency. In order to further understand the effect of electron beam irradiation, we calculated the resistivities of the AgNWs by Kirchoff’s rules given by


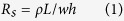


where *ρ* is the resistivity of silver, *L* is the length of the wire, *w* is the wire width and *h* is the height of the wire^19^. The resistivity of the AgNWs before electron beam irradiation (Rs = 95 ohm/sq, *L* = 10μm, *w* = *h* = 27.5 nm) and after electron beam irradiation for 120 sec (Rs = 48 ohm/sq, *L* = 10μm, *w* = *h* = 34.8 nm) was 7.2E-5 ohm.cm and 5.9E-5 ohm.cm, respectively. Clearly, reduced electron scattering by nano-soldering of the AgNW junctions after electron beam irradiation created lower resistivity in the AgNWs.

**Figure 5:**
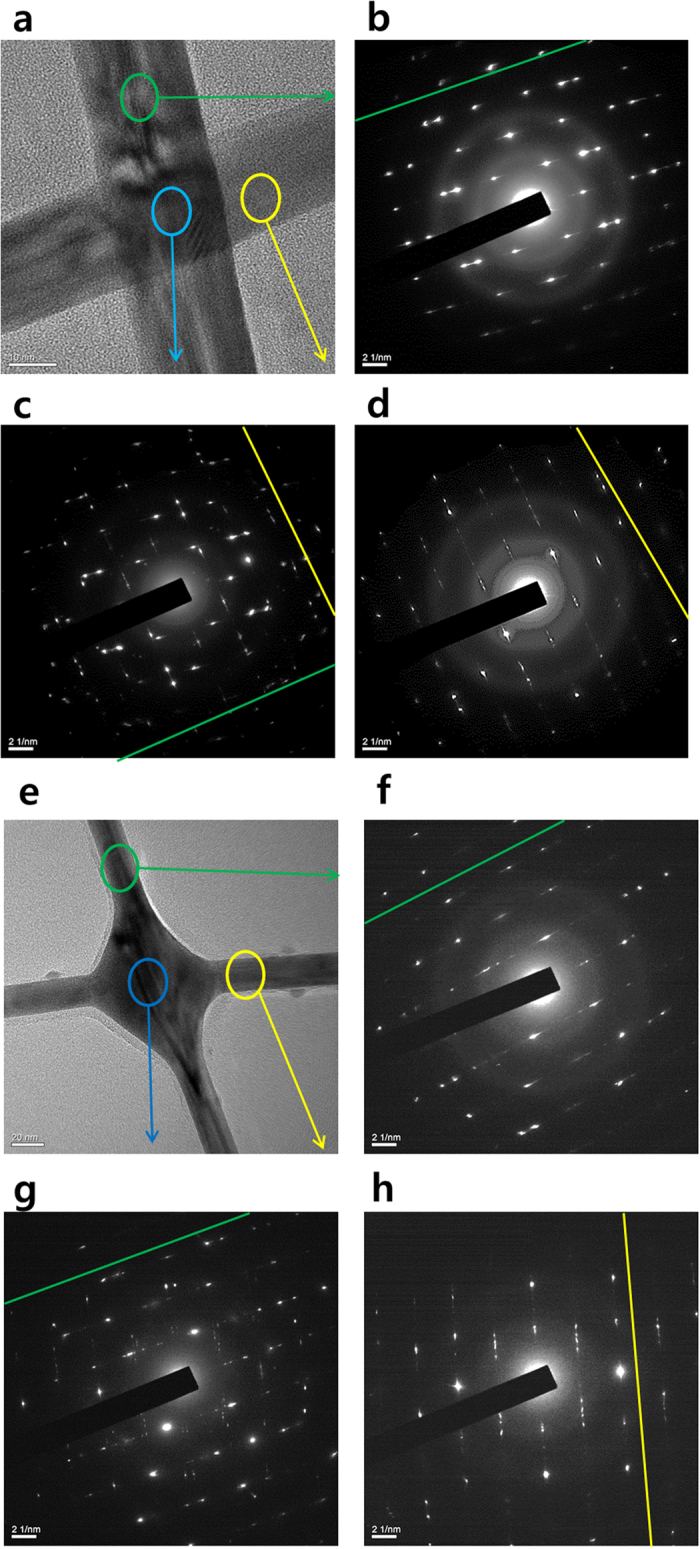
Transmission electron microscope (TEM) images of AgNW thin films before electron beam (**a**) and after electron beam (**e**), and selected area electron diffraction (SAED) of AgNW thin films before electron beam (**b–d**) and after electron beam (**f–h**).

To investigate the interfacial reactions at the AgNW junctions by electron beam irradiation, the AgNWs were examined by high resolution transmission electron microscope (TEM) and selected area electron diffraction (SAED). The AgNWs before electron beam irradiation demonstrated a pentagonally twinned nanowire crystal structure as shown in Fig. 5(b,d), as has been previously reported^21^. Figure 5(c) reveals two different crystal orientations with quite equal intensity and rotated by roughly ninety degrees at the junction point. However, after electron beam irradiation, the junctions of the AgNWs have diffraction spots, mainly along a single direction, matching the pattern from the top nanowire as shown in Fig. 5(g). Furthermore, Fig. 5(f,h) indicate that the AgNWs maintained their original crystal orientation after electron beam irradiation except for junction parts.

Thus, we demonstrate that during the electron beam irradiation on the AgNWs, the atoms in the bottom AgNWs at the junction exhibited a recrystallization, and the top nanowire acted as a nucleation template for reorientation of the bottom nanowire by electron beam irradiation. These phenomena led to developed electrical properties of the AgNWs, as has been previously reported^20^.

The substrate temperature during the electron beam process and the plasma temperature of the electron beam were less than 60 °C after 3 min and approximately 3 eV, respectively^22^. These results demonstrate that the dominant factor of the sintering of AgNW junctions may not be the thermal effect during electron beam irradiation but the high plasma temperature by energetic electrons. Thus, these energetic electrons can result in momentum transfer of electron to the junction of AgNWs, which leads to soldering of AgNWs. E. C. Garnett *et al.* and D. P. Langley *et al.* reported that local sintering at the junctions of AgNWs occurs by thermal treatment at over 150 °C (20 min) and 200 °C for 2 h, respectively^20^,^23^. The electrical and optical properties in those studies were not better than our results. However, those temperatures cannot be applied a flexible substrate. Electron beam irradiation with a low substrate temperature is more applicable for a flexible substrate than conventional thermal treatment. Furthermore, only 120 sec is needed to form the nano-soldered AgNW junctions using by electron beam irradiation. Thus, our study demonstrates that the electron beam irradiation process produces an outstanding effect on the electrical and structural properties of AgNWs with low-temperature processes for flexible applications.

**Figure 6:**
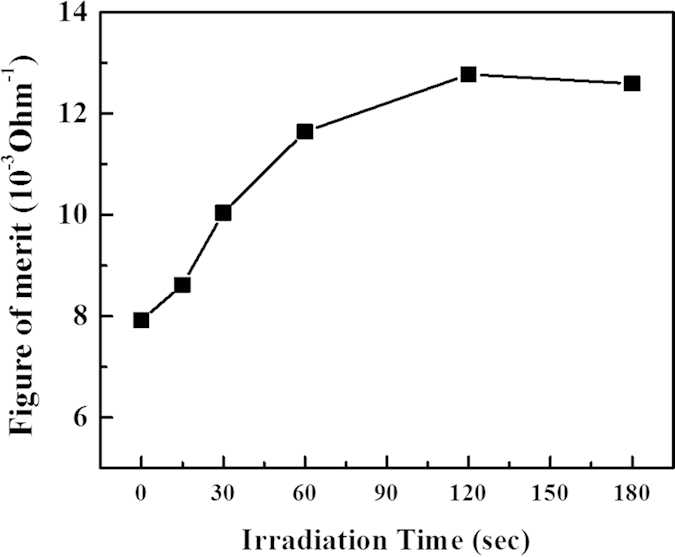
Figure of merit of AgNW thin film as a function of electron beam irradiation time.

In order to find out the best condition of AgNWs for transparent conductive material, the figure of merit (Φ_TC_) was calculated from the sheet resistance and the transmittance at a wavelength of 550 nm as shown in Fig. 5. The Φ_TC_ was defined by Haacke as in Eq. (2)^24^,


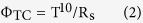


where T is the transmittance and Rs is the sheet resistance of the AgNWs. Figure 6 shows that the Φ_TC_ value of the AgNWs increased with an increase in electron beam irradiation time. The Φ_TC_ indicates similar values after electron beam irradiation for 120 and 180 sec. With these results, we can expect that AgNWs were sufficiently sintered after electron beam irradiation for 120 sec.

**Figure 7:**
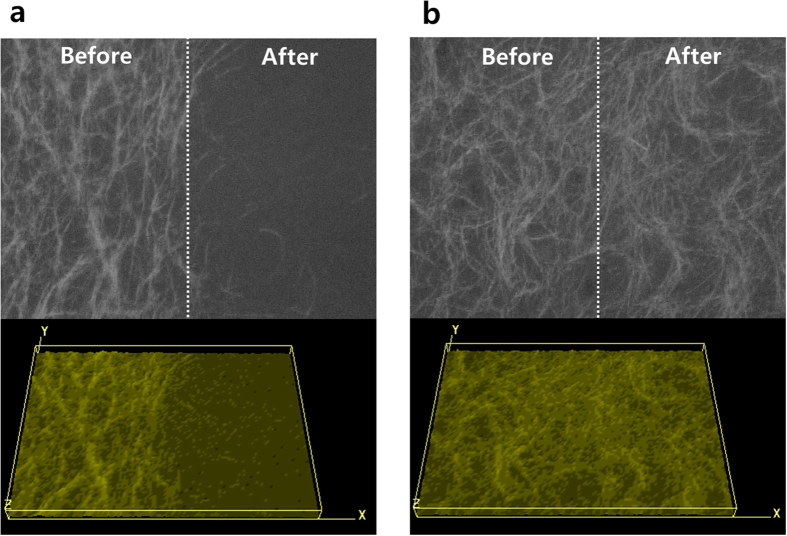
Microscope image (X100) and 3D surface plot of AgNW thin film before electron beam irradiation (**a**) and after electron beam irradiation for 120 sec (**b**) before and after 3M tape test.

To compare the adhesion of AgNWs with and without electron beam irradiation, a tape test was carried out as shown in Fig. 7. The AgNWs without electron beam irradiation were easily detached from the substrate due to the weak binding energy of the AgNWs on the glass, and the sheet resistance of detached parts increased dramatically above 500 ohm/sq. However, the AgNWs with electron beam irradiation for 120 sec exhibited an improvement of adhesion because of strong networking from soldering of the AgNWs, and the sheet resistance did not change after the taping test.

**Figure 8:**
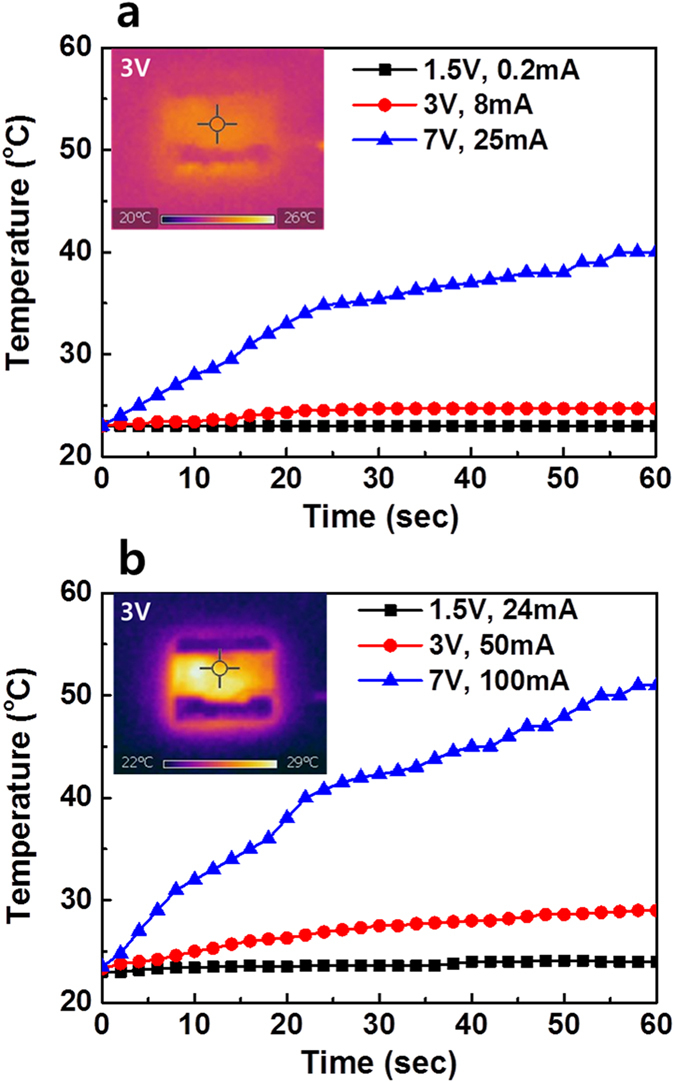
Sheet temperature as a function of heating time with different input voltages of AgNW thin film fabricated without electron beam irradiation (**a**) and with electron beam irradiation for 120 sec (**b**).

Finally, we fabricated transparent heaters using by AgNWs with and without the electron beam irradiation. In the AgNWs without electron beam irradiation, the temperature increased to 40 °C for 1 min when the input voltage was 7 V with a joule heat generation of 0.175 W as shown in Fig. 8(a). However, with AgNWs after electron beam irradiated for 120 sec, the temperature rapidly increased to 51 °C within 1 min when the input voltage was 7 V with a joule heat generation of 0.7 W as shown in Fig. 8(b). These results strongly suggest that electron beam irradiated AgNWs are a good candidate for transparent heaters such as window defrosters in cars or buildings.

## Conclusion

In this study, we demonstrated the effects of electron beam irradiation on the electrical, optical, and structural properties of AgNWs prepared by drop casting and an air-dry process. The sheet resistance of the electron beam irradiated AgNWs decreased from 95 ohm/sq (0 sec) to 48 ohm/sq (120 sec). The significantly lower sheet resistance of the AgNWs after electron beam irradiation can be attributed to nano-soldering at the junctions of the AgNWs. Furthermore, the strong networking of the AgNWs by nano-soldering improved the AgNW adhesion to the substrate. We fabricated a transparent heater using the electron beam irradiated AgNWs with low sheet resistance and high transmittance (above 88.8%). The heater temperature increased to 51 °C within 1 min at a voltage of 7 V. These results strongly suggest that AgNWs with electron beam irradiation have potential for use in transparent heater applications.

## Additional Information

**How to cite this article**: Hong, C.-H. *et al.* Electron beam irradiated silver nanowires for a highly transparent heater. *Sci. Rep.* **5**, 17716; doi: 10.1038/srep17716 (2015).
